# Selective C-Rel Activation via Malt1 Controls Anti-Fungal T_H_-17 Immunity by Dectin-1 and Dectin-2

**DOI:** 10.1371/journal.ppat.1001259

**Published:** 2011-01-20

**Authors:** Sonja I. Gringhuis, Brigitte A. Wevers, Tanja M. Kaptein, Toni M. M. van Capel, Bart Theelen, Teun Boekhout, Esther C. de Jong, Teunis B. H. Geijtenbeek

**Affiliations:** 1 Center of Infection and Immunity Amsterdam and Center for Experimental and Molecular Medicine, Academic Medical Center, University of Amsterdam, Amsterdam, The Netherlands; 2 Department of Cell Biology and Histology, Academic Medical Center, University of Amsterdam, Amsterdam, The Netherlands; 3 CBS-KNAW Fungal Biodiversity Centre, Utrecht, The Netherlands; University of Wisconsin-Madison, United States of America

## Abstract

C-type lectins dectin-1 and dectin-2 on dendritic cells elicit protective immunity against fungal infections through induction of T_H_1 and T_H_-17 cellular responses. Fungal recognition by dectin-1 on human dendritic cells engages the CARD9-Bcl10-Malt1 module to activate NF-κB. Here we demonstrate that Malt1 recruitment is pivotal to T_H_-17 immunity by selective activation of NF-κB subunit c-Rel, which induces expression of T_H_-17-polarizing cytokines IL-1β and IL-23p19. Malt1 inhibition abrogates c-Rel activation and T_H_-17 immunity to *Candida* species. We found that Malt1-mediated activation of c-Rel is similarly essential to induction of T_H_-17-polarizing cytokines by dectin-2. Whereas dectin-1 activates all NF-κB subunits, dectin-2 selectively activates c-Rel, signifying a specialized T_H_-17-enhancing function for dectin-2 in anti-fungal immunity by human dendritic cells. Thus, dectin-1 and dectin-2 control adaptive T_H_-17 immunity to fungi via Malt1-dependent activation of c-Rel.

## Author Summary

Fungal infections are a major health threat and the incidence is growing worldwide. There is a need for efficient antifungal vaccines. Adaptive immune responses and in particular T helper cell type 17 (T_H_-17) responses are crucial in the defence against fungal infections. Human dendritic cells (DCs) induce T_H_-17 responses after interaction with fungi. DCs express C-type lectins dectin-1 and dectin-2 that interact with the carbohydrate structures present in the cell-wall of fungi. It is unclear how signaling by these C-type lectins leads to specific T_H_-17 responses. Here we demonstrate that the signaling molecule Malt1 present in the CARD9-Bcl10-Malt1 complex is responsible for T_H_-17 induction by selectively activating the NF-κB transcription factor c-Rel, which drives transcription of the T_H_-17-polarizing cytokines. Inhibition of either Malt1 or c-Rel prevents T_H_-17 induction in response to fungi. Furthermore, we show that the C-type lectin dectin-2 selectively activates c-Rel, signifying a specialized T_H_-17-enhancing function for this C-type lectin. Thus, novel vaccination strategies that target dectin-2 or activate Malt1 can induce predominant T_H_-17 responses. Since aberrant T_H_-17 responses underlie the pathology of atopic dermatitis and various autoimmune diseases, Malt1 is a rational therapeutic target to attenuate anomalous adaptive immune responses.

## Introduction

Fungal infections are a major health threat and incidence of both superficial and invasive infections by *Candida* species are growing throughout the world due to increasing numbers of at-risk immunocompromised patients, such as transplant recipients and those infected with HIV-1/AIDS, as well as the emergence of strains that are resistant to antimycotic drugs [1]. Anti-fungal adaptive immunity requires both T helper cell type 1 (T_H_1) and T_H_-17 immune responses; IL-17 secreted by T_H_-17 cells mobilizes neutrophils required for anti-fungal responses [2], [3], whereas T_H_1-produced IFNγ optimally activates neutrophils and subsequent phagocytosis of fungi [4]. Dendritic cells (DCs) are crucial for the induction of T helper cell differentiation [5], [6]. Although the requirements for T_H_-17 differentiation by human DCs are not completely clear, it is evident that secretion of IL-23, IL-1β and IL-6 are important for T_H_-17 development [7], [8], whereas IL-12p70 skews T helper cell differentiation towards T_H_1 responses [9]. Little is known about the molecular mechanisms that underlie the induction of the T_H_-17-promoting cytokines by DCs after fungal infections.

Pattern recognition receptors (PRRs), such as Toll-like receptors (TLRs) and C-type lectins, sense pathogens through conserved pattern-associated molecular patterns (PAMPs), which induce signaling pathways to regulate gene transcription. C-type lectins are important in fungal recognition by DCs and in induction of anti-fungal T_H_1 and T_H_-17 immune responses [5], [10]. The cell-wall of many fungi, including *Candida* species (spp), consists of carbohydrate structures such as chitin, mannan and β-glucan that are recognized by C-type lectins like dectin-1, dectin-2, DC-SIGN and mannose receptor [5], [11], [12]. Triggering of β-glucan receptor dectin-1 by *C. albicans* induces both T_H_1 and T_H_-17 immune responses by DCs through Syk-dependent NF-κB activation [10], [13], [14]. Syk induces the assembly of a scaffold consisting of the caspase recruitment domain 9 (CARD9) protein, B cell lymphoma 10 (Bcl10) and mucosa-associated lymphoid-tissue lymphoma-translocation gene 1 (Malt1) [13], [15]. This CARD9-Bcl10-Malt1 scaffold couples dectin-1 in human to the canonical NF-κB pathway by activating NF-κB subunit p65 and c-Rel [10], [13], whereas dectin-1 triggering also leads to activation of the non-canonical NF-κB RelB pathway [10]. The balance between p65 and RelB activity is controlled by a distinct Raf-1-dependent pathway that thereby dictates expression of IL-12p70, IL-1β and IL-23 [10]. It is unclear how the CARD9-Bcl10-Malt1 complex is involved in the activation of the different NF-κB subunits and how this affects T_H_-17 differentiation. Although dectin-1-deficient people are more susceptible to mucocutaneous fungal infection, CARD9 deficiency in human causes a more pronounced phenotype with chronic mucoctaneous as well as invasive fungal infections [16], [17]. These studies suggest that dectin-1 is not the only receptor that couples CARD9-Bcl10-Malt1 to the defense against fungi. Indeed, dectin-2 interacts with *C. albicans* through mannan structures present on both yeast and hyphal forms [18], [19] and a recent study shows that dectin-2 is involved in the induction of T_H_-17 responses to *C. albicans* in mice [19], [20]. Dectin-2 indirectly activates Syk through association with the FcRγ chain [12] which results in CARD9-dependent expression of IL-2, IL-10 and TNF [20]. Thus, both dectin-1 and dectin-2 are involved in T_H_-17 development through Syk and CARD9 but the underlying mechanisms and involvement of Bcl10 and Malt1 remain unclear. It is also unclear whether dectin-1 and dectin-2 are required for a general anti-fungal response to all *Candida* species.

Here we demonstrate that dectin-1 and dectin-2 convergently contribute to anti-fungal T_H_-17 immunity by inducing IL-1β and IL-23 production. Both dectin-1 and dectin-2 triggering leads to Malt1 activation, which specifically activates NF-κB subunit c-Rel that is pivotal to the transcriptional activation of the *Il1b* and *Il23p19* genes. Syk-dependent recruitment of CARD9 and Bcl10 upon dectin-1 triggering is crucial for activation of all NF-κB subunits, while recruitment of Malt1 and activation of its paracaspase activity is distinctively required for c-Rel but not p65 or RelB activation. In contrast to dectin-1, dectin-2 triggering activates only c-Rel, which is also dependent on Malt1 signaling, signifying a specialized function for dectin-2 in T_H_-17 immunity. Simultaneous triggering of dectin-1 and dectin-2 by pathogenic fungi promotes the expression of IL-1β and IL-23 to boost T_H_-17-mediated cellular responses, whereas Malt1 inhibition after *Candida* infection markedly reduces T_H_-17 polarization. Thus, Malt1 activation links dectin-1 and dectin-2 to the c-Rel-dependent expression of IL-1β and IL-23 and directs adaptive anti-fungal immunity.

## Results

### Dectin-1 signaling via Malt1 affects IL-1β, IL-23p19, IL-6 and IL-12p35 expression

**Figure 1:**
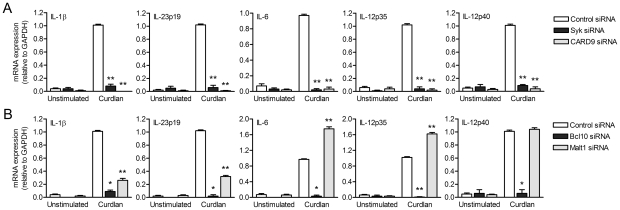
Dectin-1-induced cytokine expression requires Syk, CARD9 and Bcl10, whereas Malt1-mediated signaling enhances IL-1β and IL-23p19, but decreases IL-6 and IL-12p35 expression. (A and B) Quantitative real-time PCR for indicated mRNAs in curdlan-stimulated DCs after Syk, CARD9 (A), Bcl10 and Malt1 (B) silencing by RNA interference (siRNA). Expression is normalized to GAPDH and set at 1 in curdlan-stimulated cells. Data are mean ± s.d. of four independent experiments, *p<0.05 and **p<0.01 (Student's *t*-test).

The recruitment of the CARD9-Bcl10-Malt1 complex by Syk links dectin-1 on DCs to NF-κB activation, thereby controlling anti-fungal T_H_-17 immunity [10], [13]–[15]. In mice, the pivotal role for Syk and CARD9 in dectin-1 signaling has been established using knock-out models [13], [14], while, in contrast, little is known about their role in regulating human adaptive immunity. Here we investigated the role of the CARD9-Bcl10-Malt1 module in relaying signals from dectin-1 in human primary DCs to induce cytokine responses. We used the β-glucan curdlan, which is a specific ligand for dectin-1 and induces Syk activation in both mice and human [10], [14]. We silenced Syk, CARD9, Bcl10 and Malt1 by RNA interference (**Figure S1**) and analyzed expression of cytokines involved in T_H_1 and T_H_-17 polarization. Expression of IL-1β, IL-23p19, IL-6, IL-12p35 and IL-12p40 mRNA was completely abrogated by Syk, CARD9 as well as Bcl10 silencing (**Figure 1A and 1B**). Notably, Malt1 silencing had distinct effects on the different cytokines; IL-1β and IL-23p19 mRNA expression was strongly decreased, whereas IL-6 and IL-12p35 mRNA was enhanced and IL-12p40 mRNA expression was unaffected by Malt1 silencing (**Figure 1B**). TLR4-dependent cytokine expression was unaffected by silencing of either Syk, CARD9, Bcl10 or Malt1 (**Figure S2**). These data show that Malt1 has a very distinctive function by inducing the T_H_-17-polarizing cytokines IL-23 and IL-1β, whereas both CARD9 and Bcl10 are more generally required for all dectin-1-induced cytokine responses.

### Malt1 controls c-Rel activation by dectin-1

**Figure 2:**
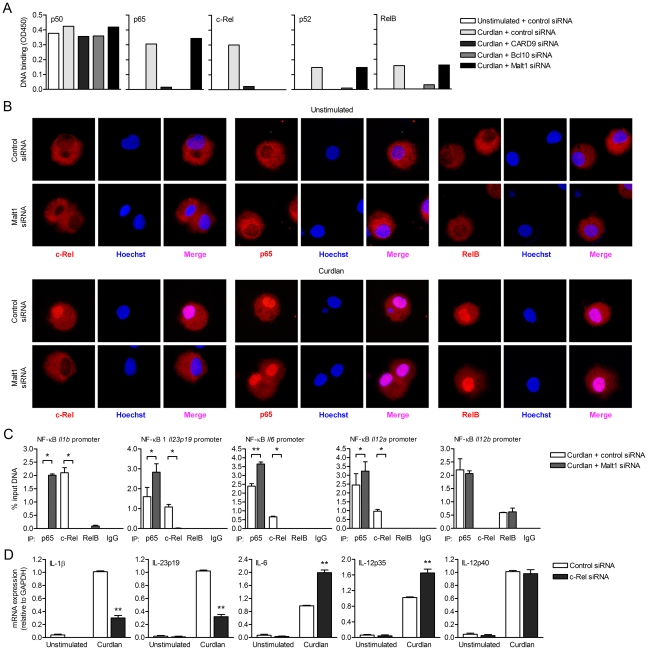
Malt1 signaling by dectin-1 is specifically required for c-Rel-dependent cytokine expression. (A) DNA binding of NF-κB subunits in nuclear extracts of curdlan-stimulated DCs after Malt1 silencing by RNA interference (siRNA). Graphs are representative of three independent experiments. (B) Translocation of c-Rel, p65 or RelB (red) into the nucleus (Hoechst staining, blue; colocalization (Merge, pink)) in curdlan-stimulated DCs after Malt1 silencing. Stainings are representative of two independent experiments. (C) ChIP assays were performed to determine binding of p65, c-Rel and RelB to NF-κB binding motifs of the *Il1b*, *Il23p19*, *Il12a*, *Il12b* and *Il6* promoters. Protein-DNA complexes were immunoprecipitated from sheared chromatin isolated from *para*-formaldehyde-fixed curdlan-stimulated DCs after Malt1 silencing by RNA interference (siRNA). Immunoprecipitation with mouse IgG served as a negative control. Quantitative real-time PCR reactions for indicated regions were performed. Levels are normalized with respect to the ‘input DNA’ sample, which had not undergone immunoprecipitation; results are expressed as the % input DNA. Data are mean ± s.d. of two independent experiments, *p<0.05 and **p<0.01 (Student's *t*-test). (D) Quantitative real-time PCR for indicated mRNAs in curdlan-stimulated DCs after c-Rel silencing by RNA interference (siRNA). Expression is normalized to GAPDH and set at 1 in curdlan-stimulated cells. Data are mean ± s.d. of four independent experiments, **p<0.01 (Student's *t*-test).

The distinct functions of CARD9, Bcl10 and Malt1 in cytokine induction after dectin-1 triggering led us to investigate their functions in the activation of NF-κB. Dectin-1 triggering activates all NF-κB subunits in a Syk-dependent manner, which is crucial to dectin-1-induced cytokine responses [10]. We first determined nuclear translocation and subsequent DNA binding of the different subunits after dectin-1 triggering. NF-κB dimers are normally retained inactive in the cytoplasm and translocate into the nucleus upon activation [21]. In control-silenced DCs, dectin-1 triggering by curdlan resulted in activation of p65, c-Rel, p52 and RelB, while p50 DNA binding could already be detected in unstimulated cells (**Figure 2A**). Silencing of either CARD9 or Bcl10 in DCs completely impaired activation of p65, c-Rel, RelB and p52 after curdlan stimulation (**Figure 2A**). Strikingly, Malt1 silencing specifically abrogated c-Rel activation (**Figure 2A**), whereas nuclear translocation of the other subunits was unaffected (**Figure 2A**). Immunofluorescence stainings showed that Malt1 silencing interfered with the nuclear translocation of c-Rel but neither with p65 nor RelB (**Figure 2B**). These data strongly suggest that Malt1 is required for selective activation of c-Rel-containing NF-κB dimers, whereas recruitment of CARD9 and Bcl10 are an absolute requirement for activation of all NF-κB subunits.

### c-Rel activation by Malt1 induces T_H_-17-polarizing cytokines IL-1β and Il-23

We next used chromatin immunoprecipitation (ChIP) assays to investigate the effect of Malt1-induced c-Rel activation on the DNA binding of the NF-κB subunits to different cytokine promoters. Our data show that the NF-κB site of the *Il1b* promoter was solely occupied by c-Rel, while both c-Rel and p65 were bound to the *Il23*, *Il6* and *Il12a* promoters after curdlan stimulation of control-silenced DCs, albeit in different ratios (**Figure 2C**). Malt1 silencing completed abrogated binding of c-Rel to the *Il1b*, *Il23*, *Il6* and *Il12a* promoters after dectin-1 triggering (**Figure 2C**), consistent with a pivotal role for Malt1-mediated signaling in c-Rel activation. The absence of c-Rel activation allowed binding of p65 to the promoters as is evident from the higher p65 association with the different promoters (**Figure 2C**). Notably, c-Rel binding to the *Il1b* promoter was completely replaced by p65 binding after Malt1 silencing (**Figure 2C**). This suggests that the *Il1b* promoter is preferentially bound by c-Rel and that c-Rel is a stronger activator of *Il1b* transcription than p65, since c-Rel replacement by p65 after Malt1 silencing resulted in significantly reduced IL-1β expression (**Figure 1B**). While Malt1 silencing abolished c-Rel binding to both the *Il23* and *Il12a* promoter after dectin-1 triggering, loss of c-Rel activation had opposite effects on *Il23* and *Il12a* transcription as IL-23p19 mRNA levels were severely decreased, while IL-12p35 mRNA was enhanced (**Figure 1B**). These results are consistent with our previous findings showing that c-Rel is a stronger transactivator of *Il23* but a weaker transactivator of *Il12a* transcripton than p65 [10]. IL-6 expression was enhanced after Malt1 silencing (**Figure 1B**), suggesting that c-Rel functions as an inhibitory factor when bound to the *Il6* promoter. The *Il12b* promoter was not bound by c-Rel in either control- or Malt1-silenced cells after curdlan stimulation (**Figure 2C**), consistent with the similar IL-12p40 mRNA levels in both control- and Malt1-silenced cells after dectin-1 triggering (**Figure 1B**).

In order to further demonstrate the importance of c-Rel in the transcriptional regulation of the *Il1b*, *Il23*, *Il6* and *Il12a* genes, we measured cytokine expression in c-Rel-silenced DCs (**Figure S1**) after curdlan stimulation (**Figure 2D**). Similar to Malt1 silencing, c-Rel silencing strongly decreased IL-1β and IL-23p19 mRNA, while enhancing IL-6 and IL-12p35 mRNA levels compared to control-silenced cells after dectin-1 triggering (**Figure 2D**). IL-12p40 mRNA expression was independent of c-Rel activation (**Figure 2D**), as was LPS-induced cytokine expression (**Figure S2**). These results strongly suggest that Malt1-mediated c-Rel activation leads to the expression of IL-1β and IL-23, key cytokines in T_H_-17 differentiation.

### Malt1 proteolytic activity is required for dectin-1-induced c-Rel-dependent cytokine expression

**Figure 3:**
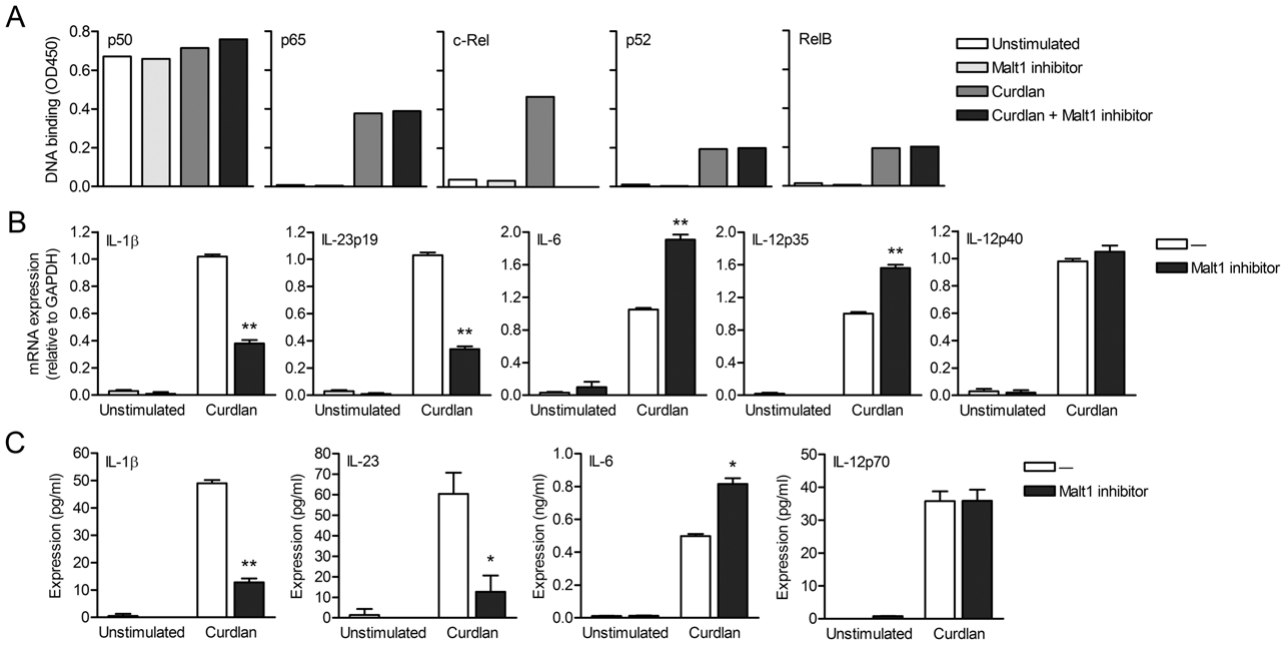
Malt1 paracaspase activity is required for c-Rel activation and cytokine induction by dectin-1. (A) DNA binding of NF-κB subunits in nuclear extracts of curdlan-stimulated DCs after inhibition of Malt1 paracaspase activity by z-VRPR-FMK. Data are representative of three independent experiments. (B) Quantitative real-time PCR for indicated mRNAs in curdlan-stimulated DCs after Malt1 paracaspase inhibition. Expression is normalized to GAPDH and set at 1 in curdlan-stimulated cells. Data are mean ± s.d. of three independent experiments, **p<0.01 (Student's *t*-test). (C) Cytokine production was determined by ELISA in supernatants of DCs stimulated with curdlan in the absence or presence of Malt1 paracaspase inhibitor. Data are mean ± s.d. of duplicate cultures, and are representative of five independent experiments, *p<0.05 and **p<0.01 (Student's *t*-test).

Since Malt1 has paracaspase activity [22], [23], we next investigated whether the adaptor or protease function of Malt1 is involved in the selective activation of c-Rel after dectin-1 triggering. We used z-VRPR-FMK, a compound which blocks the proteolytic activity of Malt1 [22]. Inhibition of Malt1 proteolytic activity completely abolished activation of c-Rel without affecting the other NF-κB subunits (**Figure 3A**), which is similar to Malt1 silencing (**Figure 2A**), Immunofluoresence stainings confirmed that Malt1 inhibition specifically interferes with nuclear translocation of c-Rel after curdlan stimulation (**Figure S3**). Malt1 paracaspase inhibition also markedly reduced both IL-1β and IL-23p19 mRNA levels and slightly enhanced IL-6 and IL-12p35 mRNA production after curdlan stimulation (**Figure 3B**), similarly to Malt1 silencing (**Figure 1B**). IL-12p40 mRNA production was neither dependent on Malt1 expression nor activation (**Figure 3B**). We next measured cytokine production and found that Malt1 inhibition severely reduced IL-1β and IL-23 protein expression, without affecting IL-12p70 expression and only slightly enhancing IL-6 expression (**Figure 3C**), indicating that dectin-1-induced cytokine expression is primarily regulated at the transcriptional level. These results show that Malt1 protease activity is required for specific c-Rel activation and plays a central role in the induction of T_H_-17-polarizing cytokines by dectin-1.

### Malt1 directs expression of T_H_-17-polarizing cytokines during *Candida* spp. infection

**Figure 4:**
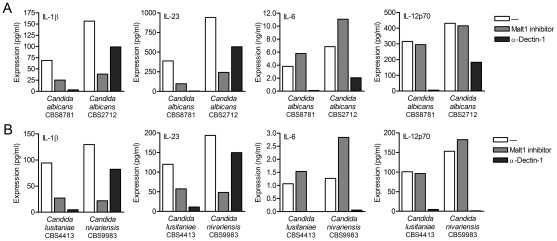
Malt activation controls IL-1β and IL-23 production in response to *Candida* spp. (A and B) Cytokine production was determined by ELISA in supernatants of DCs stimulated with *Candida albicans* spp. (A), *C. nivariensis* or *C. lusitaniae* (B) in the absence or presence of Malt1 paracaspase inhibitor z-VRPR-FMK or blocking dectin-1 antibodies. Data are representative of three independent experiments.

Dectin-1 plays an important role in anti-fungal immunity through the induction of T_H_1 and T_H_-17 differentiation [10], [14]. Since *Candida albicans* infections are amongst the most common causes of invasive fungal infections in immunocompromised patients [24], [25], we used two different *C. albicans* strains to investigate the importance of Malt1 signaling in anti-fungal immune responses. Consistent with its function in the induction of T_H_-17-polarizing cytokines, Malt1 activation is required for expression of IL-1β and IL-23 by DCs in response to both *C. albicans* strain CBS8781 and CBS2712 (**Figure 4A**). As observed with curdlan stimulation, the expression of IL-6 was slightly upregulated as a result of Malt1 protease inhibition, whereas IL-12p70 production was unaffected by Malt1 signaling after *C. albicans* stimulation (**Figure 4A**). To elucidate the contribution of dectin-1 signaling to the Malt1-dependent cytokines responses, we treated DCs with *C. albicans* in the presence of blocking dectin-1 antibodies. Notably, we observed that *C. albicans* CBS8781-induced cytokine expression was completely abrogated after blocking dectin-1, whereas cytokine production after *C. albicans* CBS2712 stimulation was only partially inhibited by dectin-1 antibodies (**Figure 4A**). Malt1 inhibition decreased *C. albicans* CBS2712-induced IL-1β and IL-23 expression more strongly than dectin-1 inhibition (**Figure 4A**). These results suggest that fungal infections trigger not only dectin-1 but also other receptors to induce anti-fungal T_H_-17 responses via Malt1. To further investigate this, we used two different *Candida* species, *C. lusitaniae* and *C. nivarienis*, both emerging pathogenic fungi causing opportunistic infections in transplant and immunocompromised patients [24], [26]. *C. lusitaniae* CBS4413 induced cytokine production in a dectin-1-dependent manner, while *C. nivariensis* CBS9983 was only partially dependent on dectin-1 signaling for the production of IL-1β, IL-23, IL-6 and IL-12p70 (**Figure 4B**). Both IL-1β and IL-23 production by *C. lusitaniae* and *C. nivariensis* was largely dependent on Malt1 protease activity (**Figure 4B**). Noteworthy, *Candida* spp. that trigger Malt1 activation via dectin-1 in combination with other unidentified receptor(s) induce higher levels of IL-1β and IL-23 in DCs than those that trigger only dectin-1 (**Figure 4A and 4B**). Similar to the *C. albicans* strains, *C. lusitaniae* and *C. nivariensis* stimulation resulted in slightly enhanced IL-6 expression after Malt1 inhibition, while IL-12p70 was unaffected (**Figure 4B**). These data suggest that c-Rel activation by Malt1 signaling controls anti-fungal T_H_-17 immunity to *Candida* spp.

### Dectin-2 contributes to cytokine response during *Candida* spp. infection

**Figure 5:**
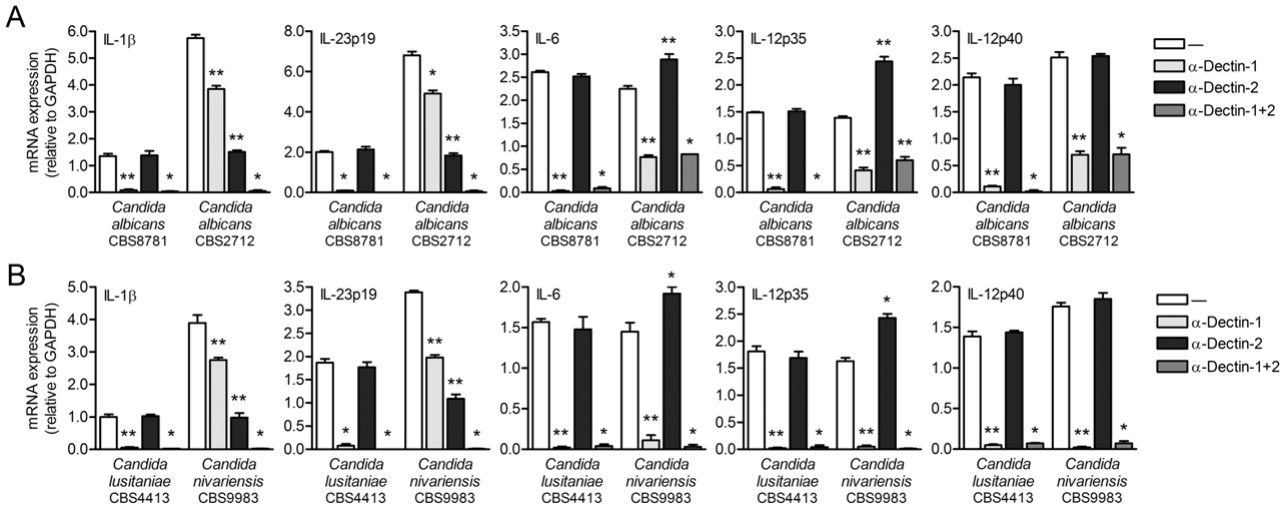
Dectin-1 and dectin-2 contribute to *Candida* spp.-induced cytokine expression. (A and B) Quantitative real-time PCR for indicated mRNAs in DCs stimulated with *Candida albicans* spp. (A), *C. nivariensis* or *C. lusitaniae* (B) in the absence or presence of blocking antibodies against dectin-1 and/or dectin-2. Expression is normalized to GAPDH and set at 1 in curdlan-stimulated cells. Data are mean ± s.d. of four independent experiments, *p<0.05 and **p<0.01 (Student's *t*-test).

We next set out to identify the fungal PRR(s) on DCs that are triggered by *C. albicans* CBS2712 and *C. nivariensis* to induce Malt1 activation independently of dectin-1. The C-type lectin dectin-2 has been shown to participate in fungal T_H_-17 immunity in mice [19], [20]. We explored a role for dectin-2 in cytokine responses to *Candida* spp. by using blocking antibodies against dectin-2. Notably, induction of IL-1β and IL-23p19 mRNA by both *C. albicans* CBS2712 and *C. nivariensis* was partially abolished by dectin-2 antibodies, while blocking both dectin-1 and dectin-2 completely abrogated expression of IL-1β and IL-23p19 (**Figure 5**). These data strongly suggest that dectin-2 signaling contributes together with dectin-1 to the induction of these T_H_-17-polarizing cytokines by *C. albicans* CBS2712 and *C. nivariensis*. Blocking dectin-2 triggering by *C. albicans* CBS2712 or *C. nivariensis* slightly increased IL-6 but greatly enhanced IL-12p35 mRNA expression (**Figure 5**), most likely reflecting the negative influence of c-Rel binding to the respective promoters on *Il6* and *Il12a* transcription. *C. nivariensis*-induced IL-6 and IL-12p35 mRNA expression was dependent on both dectin-1 and dectin-2, while *C. albicans* CBS2712 induced IL-6 and IL-12p35 expression via dectin-1, as blocking both dectin-1 and dectin-2 had no additional effects compared to blocking dectin-1 alone (**Figure 5**). IL-12p40 mRNA expression by *C. albicans* CBS2712 and *C. nivariensis* was independent of dectin-2 triggering (**Figure 5**). These data suggest that dectin-2 signaling through Malt1 controls only c-Rel-dependent gene expression without affecting c-Rel-independent transcription. The residual expression of IL-6, IL-12p35 and IL-12p40 induced by *C. albicans* CBS2712 after either blocking dectin-1 or dectin-1 plus dectin-2 suggests additional involvement of other receptors, such as TLRs (**Figure 5A**). As expected, dectin-2 antibodies did not interfere with cytokine expression induced by *C. albicans* CBS8781 and *C. lusitaniae*, since cytokine induction was completely inhibited by blocking dectin-1 antibodies (**Figure 4** **and** **5**). Cytokine protein levels confirmed the mRNA expression data (**Figure S4**). Our data demonstrate that both dectin-1 and dectin-2 contribute to anti-fungal T_H_-17-polarizing cytokine responses to various *Candida* spp.

### Malt1 relays dectin-2 signals to induce c-Rel-dependent cytokine expression

**Figure 6:**
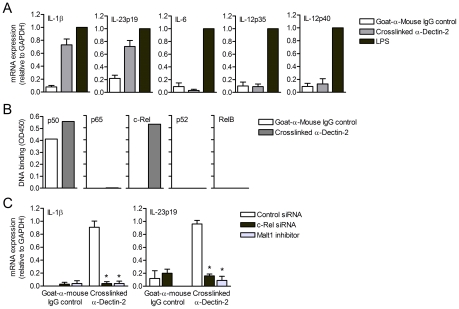
Dectin-2 signaling induces Malt1- and c-Rel-dependent IL-1β and IL-23p19 expression. (A) Quantitative real-time PCR for indicated mRNAs in DCs stimulated with crosslinked dectin-2 antibodies or LPS. In (A–C), goat-anti-mouse IgG was coated as a control for aspecific activation. Expression is normalized to GAPDH and set at 1 in LPS-stimulated cells. Data are mean ± s.d. of at least four independent experiments. (B) DNA binding of NF-κB subunits in nuclear extracts of dectin-2-triggered DCs. Data are representative of two independent experiments. (C) Quantitative real-time PCR for indicated mRNAs in DCs stimulated with crosslinked dectin-2 antibodies after c-Rel silencing or Malt1 inhibition with paracaspase inhibitor z-VRPR-FMK. Goat-anti-mouse IgG was coated as a control for non-specific activation. Expression is normalized to GAPDH and set at 1 in LPS-stimulated cells. Data are mean ± s.d. of two independent experiments, *p<0.05 (Student's *t*-test).

We next triggered dectin-2-FcRγ signaling by crosslinking dectin-2 with antibodies and investigated cytokine expression induced by human DCs. Dectin-2 crosslinking induced high levels of IL-1β and IL-23p19 mRNA expression (**Figure 6A**). Remarkably, dectin-2 crosslinking did neither induce IL-6, IL-12p35 nor IL-12p40 mRNA expression (**Figure 6A**), consistent with our observations when blocking dectin-2 binding by *Candida* spp. (**Figure 5**) and strongly suggesting that dectin-2 triggering specifically induces IL-1β and IL-23p19. These results confirm that dectin-1 and dectin-2 signaling converge to boost the expression of T_H_-17-polarizing cytokines, as we observed after *Candida* spp. stimulation.

We next investigated whether dectin-2 crosslinking induces NF-κB activation. Notably, dectin-2 triggering resulted in the specific activation of c-Rel, whereas the other NF-κB subunits p65, RelB and p52 were not activated (**Figure 6B**). Consistently, c-Rel-silenced DCs exhibited a defect in the induction of IL-1β and IL-23p19 mRNA expression after dectin-2 crosslinking (**Figure 6C**). We next silenced Malt1 expression to investigate whether dectin-2-FcRγ signaling, like dectin-1, employs Malt1 to specifically activate c-Rel and induce c-Rel-dependent cytokine expression. Similar to c-Rel silencing, Malt1 silencing completely abolished IL-1β and IL-23p19 mRNA production in response to dectin-2 crosslinking (**Figure 6C**). These data demonstrate that dectin-2 has a specialized function in adaptive immunity and specifically contributes to the induction of IL-1β and IL-23p19, emphasizing the importance of the Malt1-c-Rel activation axis in T_H_-17 immunity.

### Malt1 signaling skews T helper cell polarization towards T_H_-17

**Figure 7:**
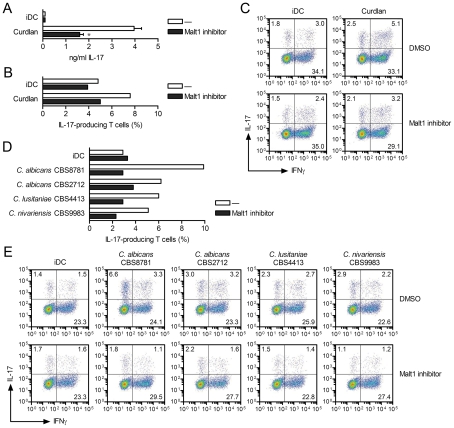
Malt1 signaling skews T helper cell polarization towards T_H_-17. (A–E) T helper cell polarization was assessed by ELISA by measuring IL-17 production in supernatants at day 5 (A) or by flow cytometry by staining for intracellular IL-17 or IFNγ expression at day 12 after PMA plus ionomycin restimulation (B–E), after co-culture of memory CD4^+^ T cells with DCs left unstimulated (iDC) or primed with curdlan (A–C) or *Candida* spp. (D and E) in the absence or presence of Malt1 paracaspase inhibitor z-VRPR-FMK. In (B) and (D) the percentage of IL-17-producing T cells are shown, corresponding to the upper left and right quadrants of (C) and (E), respectively. Data are mean ± s.d. of duplicate cultures, *p<0.05 (Student's *t*-test), and are representative of three (A) or two (B–E) independent experiments.

Since Malt1 links dectin-1 and dectin-2 to the expression of the T_H_-17-polarizing cytokines IL-1β and IL-23 via the activation of c-Rel, we investigated whether Malt1 activation affects adaptive immunity to *Candida* spp. We first co-cultured curdlan-primed DCs with CD4^+^ T cells and measured IL-17 secretion after 5–12 days of co-culture [7]. Malt1 inhibition markedly reduced the capacity of curdlan-primed DCs to induce IL-17 expression in CD4^+^ T cells (**Figure 7A, 7B and 7C**). Thus, Malt1 activity is essential for the induction of T_H_-17-polarizing cytokines in DCs via dectin-1 triggering and subsequent T_H_-17 skewing. The ability of DCs primed by the different *Candida* spp. to promote IL-17 expression in CD4^+^ T cells was completely blocked when Malt1 activation was inhibited in the DCs (**Figure 7D and 7E**). This effect of Malt1 inhibition on the ability of *Candida*-primed DCs to induce T_H_-17 polarization was irrespective of the involvement of either dectin-1 alone (*C. albicans* CBS8781 and *C. lusitaniae*) or combined dectin-1 and dectin-2 triggering (*C. albicans* CBS2712 and *C. nivariensis*), consistent with a general role for Malt1 in inducing the T_H_-17-polarizing cytokines IL-1β and IL-23. Thus, the marked impact of Malt1 inhibition on IL-1β and IL-23p19 expression in response to fungal infections translates to a block in T_H_-17 immunity. Our data demonstrate that Malt1 signaling to c-Rel activation drives anti-fungal T_H_-17 responses.

## Discussion

C-type lectins are amongst the most important innate receptors on DCs to induce anti-fungal T_H_-17 immunity [5], [11], [14]. Expression of cytokines upon dectin-1 triggering by fungi requires NF-κB activation through Syk-dependent CARD9-Bcl10-Malt1 signaling [13], [15]. Here we demonstrate that Malt1 activation by dectin-1 and dectin-2 on human DCs induces the expression of T_H_-17-polarizing cytokines IL-1β and IL-23 through selective activation of the NF-κB subunit c-Rel. c-Rel is crucial for optimal transcription of the *Il1b* and *Il23p19* genes. Dectin-1-induced activation of p65, RelB and c-Rel is completely dependent on the recruitment of CARD9 and Bcl10. Notably, Malt1 through its proteolytic paracaspase activity is specifically involved in activation of c-Rel, but dispensable for p65 and RelB activation. Malt1 activation of c-Rel is similarly essential in the induction of T_H_-17-polarizing cytokines by dectin-2. Strikingly, dectin-2 signaling, unlike dectin-1, only induces strong c-Rel, but not p65 and RelB activation, strongly suggestive of a specific T_H_-17 polarizing function of dectin-2. Furthermore, the involvement of dectin-1 and detcin-2 in anti-fungal immunity by human DCs depends on the *Candida* species. Our data strongly suggest that dectin-2 is crucial in recognition of some pathogenic *Candida* species to boost dectin-1-induced T_H_-17 responses via Malt1. Thus, Malt1-dependent activation of c-Rel dictates adaptive T_H_-17 immunity to fungi by dectin-1 and dectin-2.

Protective immunity against fungal infections via T_H_-17 cellular responses requires the expression of IL-1β, IL-23 and IL-6 by DCs. Here we demonstrated that selective activation of NF-κB family member c-Rel by dectin-1 and dectin-2 signaling in response to fungi was essential to expression of IL-1β and IL-23 and consequently T_H_-17 immunity. Our data showed that loss of c-Rel binding to the *Il1b* and *Il23p19* promoters strongly decreased IL-1β and IL-23p19 expression even though p65 bound to the NF-κB binding sites in the absence of c-Rel activation. These data further showed that c-Rel was the stronger activator of *Il1b* and I*l23* transcription. Furthermore, c-Rel had an inhibitory effect on *Il6* transcription, although p65-driven IL-6 expression allows for sufficient IL-6 to direct T_H_-17 polarization.

Both dectin-1 and dectin-2 triggering resulted in c-Rel activation and c-Rel-dependent IL-1β and IL-23 expression. Engagement of dectin-1 by fungal ligands leads to phosphorylation of the immunoreceptor tyrosine-based activation motif (ITAM)-like sequence within its cytoplasmic domain [15], [27] and subsequent association of the spleen tyrosine kinase Syk. Syk activation by dectin-1 is required for NF-κB activation via the assembly of the CARD9-Bcl10-Malt1 module [13], [14]. Unlike dectin-1, dectin-2 requires pairing with the adaptor molecule FcRγ to induce signaling [12], [20]. Dectin-2 triggering results in phosphorylation of the ITAM of FcRγ and activation of Syk signaling, which induces cytokine expression [20]. Dectin-2 signaling is CARD9-dependent, however a role for Bcl10 and Malt1 remains to be established [20]. In antigen receptor signaling, oligomerization of CARD11 (CARMA1) triggers the formation of a scaffold that physically bridges the CARD11-Bcl10-Malt1 complex with downstream signaling effectors, such as TRAFs and TAK1, to activate the NF-κB-regulating IKK complex [28]. Here we demonstrated that c-Rel activation by dectin-1 and dectin-2 is completely dependent on Malt1 activation. Malt1 is an unique protein as it is the only human paracaspase known [22], [23] and our data showed that its paracaspase activity was essential to the activation of c-Rel by dectin-1 and dectin-2. Malt1 has a distinctive function within the CARD9-Bcl10-Malt1 complex induced upon dectin-1 and dectin-2 triggering since silencing of CARD9 and Bcl10 by RNA interference completely abrogated the activation of all NF-κB subunits, while Malt1 silencing selectively abrogated c-Rel activation. It is unclear how Malt1 specifically activates c-Rel. A similar observation has been reported for B cell receptor signaling, which uses the CARD11-Bcl10-Malt1 complex for NF-κB activation [29], while Malt1 is involved in RelB activation after BAFF stimulation in specific B cell subsets [30]. In T cell receptor signaling, the paracaspase activity of Malt1 partially accounts for the amount of NF-κB activation [22], which might reflect the c-Rel-dependency in T cell receptor responses. Only two substrates for Malt1 are known, its binding partner Bcl10 and A20 that functions as an inhibitor of NF-κB activation [22], [23], but it remains to be determined if they have any role in the selective activation of c-Rel via Malt1. We showed that dectin-2 signaling only induced strong c-Rel activation, while dectin-1 triggering activated all NF-κB subunits; possibly the differential use of downstream molecules like TRAFs by dectin-1 and dectin-2 might underlie these differences in NF-κB activation.

Crosstalk between signaling pathways triggered by recognition of different PAMPs by various PRRs is essential to the induction of immune responses [5], [6], [11]. Here we demonstrated that dectin-1 and dectin-2 play distinct roles in immunity to fungi. While dectin-1 triggering induced cytokines involved in promoting both T_H_1 and T_H_-17 polarization, dectin-2 triggering resulted specifically in IL-1β and IL-23p19 expression, which enhanced IL-1β and IL-23 expression in response to different pathogenic *Candida* spp. This suggests that dectin-1 functions more broadly as an anti-fungal receptor inducing protective immunity, while dectin-2 is more specialized in boosting T_H_-17 cellular responses. Our data also demonstrated that even related pathogenic fungi triggered different sets of PRRs, likely contributing to tailoring of pathogen-specific immunity. *C. albicans* strain CBS8781 and *C. lusitaniae* induced cytokine expression in a dectin-1-dependent manner. In contrast, *C. albicans* strain CBS2712 and *C. nivariensis* triggered both dectin-1 and dectin-2 and showed higher IL-1β and IL-23 responses, strongly suggesting that dectin-1 and dectin-2 signaling pathways converge to enhance T_H_-17 immunity. Other *Candida* species might preferentially trigger dectin-2 but not dectin-1 for IL-1β and IL-23p19 protein expression as shown in the study of Saijo *et al.* [19]. Notably, *C. albicans* CBS2712 also induced dectin-1- and dectin-2-independent expression of IL-6, IL-12p35 and IL-12p40. The contribution of TLR signaling, especially via TLR2, and collaboration with dectin-1 signaling has previously been recognized in cytokine responses in *Candida* infections [11], [31]. However, C-type lectin triggering seems to be more specialized in IL-23p19 and IL-1β induction. The situation in mice might be more complex as murine TLRs seem to induce c-Rel activation [32], while human TLRs do not [33]. Our data emphasize that immune responses are tailored not only to pathogens from different species but even within species. Thus, interpretation of data obtained with a single pathogen should be done with caution. Research into the role of dectin-1 in fungal infections using knock-out mice has resulted in conflicting data [34], [35] and the use of different yet related fungi might underlie these differences. Genetic variation within the *Candida* clade might not only account for differences in pathogenicity [36] but also for the differential recognition by innate receptors. We have demonstrated here that even closely related *C. albicans* strains trigger different sets of PRRs to activate adaptive immune responses.

Malt1-mediated c-Rel activation might be a general mechanism for induction of protective T_H_-17 immunity against fungi and other microbes, since the Card9-Bcl10-Malt1 complex might couple other C-type lectins besides dectin-1 and dectin-2 to NF-κB activation. Furthermore, the carbohydrate specificities of dectin-1 and dectin-2 for β-glucans and high mannoses, respectively, signify their importance in more general anti-fungal immunity against species from the phylum *Ascomycota* that contain mannan, chitin and glucan structures in their cell-wall [37], [38]. Many pathogenic ascomycetes such as *Candida* spp., *Aspergillus* spp., *Coccidiodides* spp., *Pneumocystis jirovecii* (previously known as *Pneumocystis carinii*), *Histoplasma capsulatum*, *Trichophyton rubrum* and *Microsporum audouinni* have been identified as dectin-1 and/or dectin-2 ligands [12], [20], [34], [35], [39]–[41]. In contrast, the cell-wall of fungi from the phylum *Basidiomycota*, such as *Cryptococcus* and *Malassezia* spp, differs from that of ascomycetes, as it is enfolded in a glucuronic acid-rich carbohydrate capsule or consists of lipophilic structures, respectively [42], [43]. T_H_-17 responses to *Cr. neoformans* have been reported [44] but are not mediated by dectin-1 [45].

Thus, dectin-1 and dectin-2 control c-Rel activation distinctively via Malt1 activation to induce IL-1β and IL-23 expression and as such tailor T_H_-17 immune responses against fungal pathogens. Given the pivotal role of T_H_-17 responses not only in protective immunity against fungi but also in the pathology of human autoimmune diseases like Crohn's disease, ulcerative colitis, psoriasis and in vaccine development against tuberculosis [3], [46], our results might benefit therapeutic developments as Malt1 presents a rational target for immunomodulatory drugs.

## Materials and Methods

### Cells, stimulation, inhibition and RNA interference

This study was performed in accordance with the ethical guidelines of the Academic Medical Center. Immature DCs (iDC, day 6 and 7) were generated as described previously [10]. DCs were stimulated with 10 µg/ml curdlan (Sigma), heat-killed *Candida* spp. [47] (multiplicity of infection (MOI) 10) and 10 ng/ml LPS from *Salmonella typhosa* (Sigma). Dectin-2 triggering was induced by pre-incubating DCs for 2 h at room temperature with 5 µg/ml anti-dectin-2 (MAB3114; R&D Systems), followed by crosslinking on goat-anti-mouse IgG (115-006-0710; Jackson)-coated culture plates. Cells were preincubated with blocking antibodies or inhibitor for 2 h with 20 µg/ml anti-dectin-1 (MAB1859; R&D Systems), 20 µg/ml anti-dectin-2 (MAB3114; R&D Systems) or 75 µM z-VRPR-FMK (Malt1 inhibitor [22]; Alexis). DCs were transfected with 25 nM siRNA using transfection reagent DF4 (Dharmacon), and used for experiments 72 h after transfection. ‘SMARTpool’ siRNAs used were: Syk (M-003176-03), CARD9 (M-004400-01), Bcl10 (M-004381-02), Malt1 (M-005936-02), c-Rel (M-004768-01) and non-targeting siRNA (D-001206-13) as a control (Dharmacon). This protocol resulted in nearly 100% transfection efficiency as determined by flow cytometry of cells transfected with siGLO-RISC free-siRNA (D-001600-01) and did not induce IFN responses as determined by quantitative real-time PCR analysis [10]. Silencing of expression was verified by real-time PCR and flow cytometry (**Figure S1**).

### Quantitative real-time PCR

mRNA isolation, cDNA synthesis and PCR amplification with the SYBR green method in an ABI 7500 Fast PCR detection system (Applied Biosystems) were performed as described [10]. Specific primers were designed using Primer Express 2.0 (Applied Biosystems; **Table S1**). The C_t_ value is defined as the number of PCR cycles where the fluorescence signal exceeds the detection threshold value. For each sample, the normalized amount of target mRNA was calculated from the obtained C_t_ values for both target and GAPDH mRNA with N_t_ = 2^Ct(GAPDH)−Ct(target)^. The relative mRNA expression was obtained by setting N_t_ in curdlan- or LPS-stimulated samples at 1 within one experiment and for each donor.

### Cytokine production

Cell culture supernatants were harvested after 28 h of stimulation and concentrations of IL-1β, IL-23, IL-6 (Invitrogen) and IL-12p70 (eBioscience) were determined by ELISA.

### Chromatin immunoprecipitation (ChIP) assay

ChIP assays were performed using the ChIP-IT Express Enzymatic kit (Active Motif) to determine occupancy of the regulatory regions of the *Il1b*, *Il23p19*, *Il6*, *Il12a* and *Il12b* promoters by NF-κB as described by the manufacturer. Protein/DNA complexes were immunoprecipitated using anti-p65 (3034; Cell Signaling), anti-c-Rel (4727; Cell Signaling), anti-RelB (4954; Cell Signaling) or negative control IgG (53010; Active Motif), and protein G-coated magnetic beads. DNA was purified after reversal of crosslinks and real-time PCR reactions were then performed with primer sets spanning the NF-κB binding sites (**Table S1**). Primers spanning genomic DNA at cytogenetic location 12 p13.3 (Active Motif) were used as a negative control. To normalize for DNA input, a sample for each condition was taken along which had not undergone immunoprecipitation with a specific antibody (‘input DNA’); the results are expressed as the % input DNA.

### Immunofluorescence staining

Stainings were performed as described previously [10] with with anti-p65, anti-c-Rel or anti-RelB (all from Cell Signaling) followed by Alexa Fluor 594-conjugated goat anti-rabbit (A11072; Molecular Probes).

### NF-κB DNA binding

Nuclear extracts of DCs were prepared using NucBuster protein extraction kit (Novagen) and NF-κB DNA binding determined using TransAM NF-κB family kit (Active Motif).

### T_H_-17 polarization assay

Memory CD4^+^ T cells were isolated as described previously [7]. iDCs were preincubated for 2 h with inhibitors, activated for 16 h with curdlan or heat-killed *Candida* spp. and subsequently co-cultured with memory CD4^+^ T cells as described (20,000 T cells/2000 DCs in the presence of 10 pg/ml *Staphylococcus aureus* enterotoxin B (Sigma) [7]. After 5 days of co-culture, supernatants were harvested and analyzed for IL-17 production by ELISA (Biosource). Cells were further cultured in the presence of 10 U/ml IL-2 (Chiron) and resting cells were restimulated after 12 days with 100 ng/ml PMA (Sigma) and 1 µg/ml ionomycin (Sigma) for 6 h, the last 5 h in the presence of 10 µg/ml brefeldin A (Sigma), and analyzed for intracellular cytokine expression by staining with biotinylated mouse anti-IL-17 (ebio64DE; eBioscience), followed by incubation with streptavidin-PE (BD Pharmingen) and FITC-conjugated mouse anti-IFN-γ (25723.11; BD).

### Statistical analysis

Student's *t*-test for paired observations was used for statistical analyses. Statistical significance was set at a *P* values of less than 0.05.

## Supporting Information

Figure S1 Silencing of Syk, CARD9, Bcl10, Malt1 and c-Rel in human primary DCs by RNA interference. Indicated proteins were silenced using specific SMARTpools, and non-targeting siRNA as a control. Silencing was confirmed by quantitative real-time PCR (A, C, E, G and I), or by staining and flow cytometry (B, D, F, H and J). In (A, C, E, G and I), expression is normalized to GAPDH and set at 1 in control siRNA-treated cells. Data are mean ± s.d. of at least four independent experiments (A, C, E, G and I) or are representative of at least two independent experiments (B, D, F, H and J).
(2.24 MB TIF)

Figure S2 LPS signaling is not affected by Syk, CARD9, Bcl10, Malt1 and c-Rel silencing. Quantitative real-time PCR of indicated mRNAs in curdlan-stimulated DCs after Syk, CARD9, Bcl10, Malt1 and c-Rel silencing by RNA interference (siRNA). Expression is normalized to GAPDH and set at 1 in curdlan-stimulated cells. Data are mean ± s.d. of at least three independent experiments.
(2.22 MB TIF)

Figure S3 Malt1 paracaspase activity is required for c-Rel activation by dectin-1. Translocation of c-Rel, p65 or RelB (red) into the nucleus (Hoechst staining, blue; colocalization (Merge, pink)) in curdlan-stimulated DCs after Malt1 paracaspase inhibition by z-VRPR-FMK. Stainings are representative of two independent experiments.
(7.54 MB TIF)

Figure S4 Dectin-1 and dectin-2 contribute to Candida spp.-induced cytokine expression. Cytokine production was determined by ELISA in supernatants of DCs stimulated with Candida albicans spp. (A), C. nivariensis or C. lusitaniae (B) in the absence or presence of blocking antibodies against dectin-1 and/or dectin-2. Data are representative of two independent experiments.
(2.36 MB TIF)

Table S1 Expression primer sequences.
(0.05 MB DOC)
