# Supplementary figures and images for: Selective C-Rel Activation via Malt1 Controls Anti-Fungal TH-17 Immunity by Dectin-1 and Dectin-2
Source: PLoS Pathog. 2011 Jan 20;7(1):e1001259. doi: 10.1371/journal.ppat.1001259 (PMC3024268; doi:10.1371/journal.ppat.1001259)

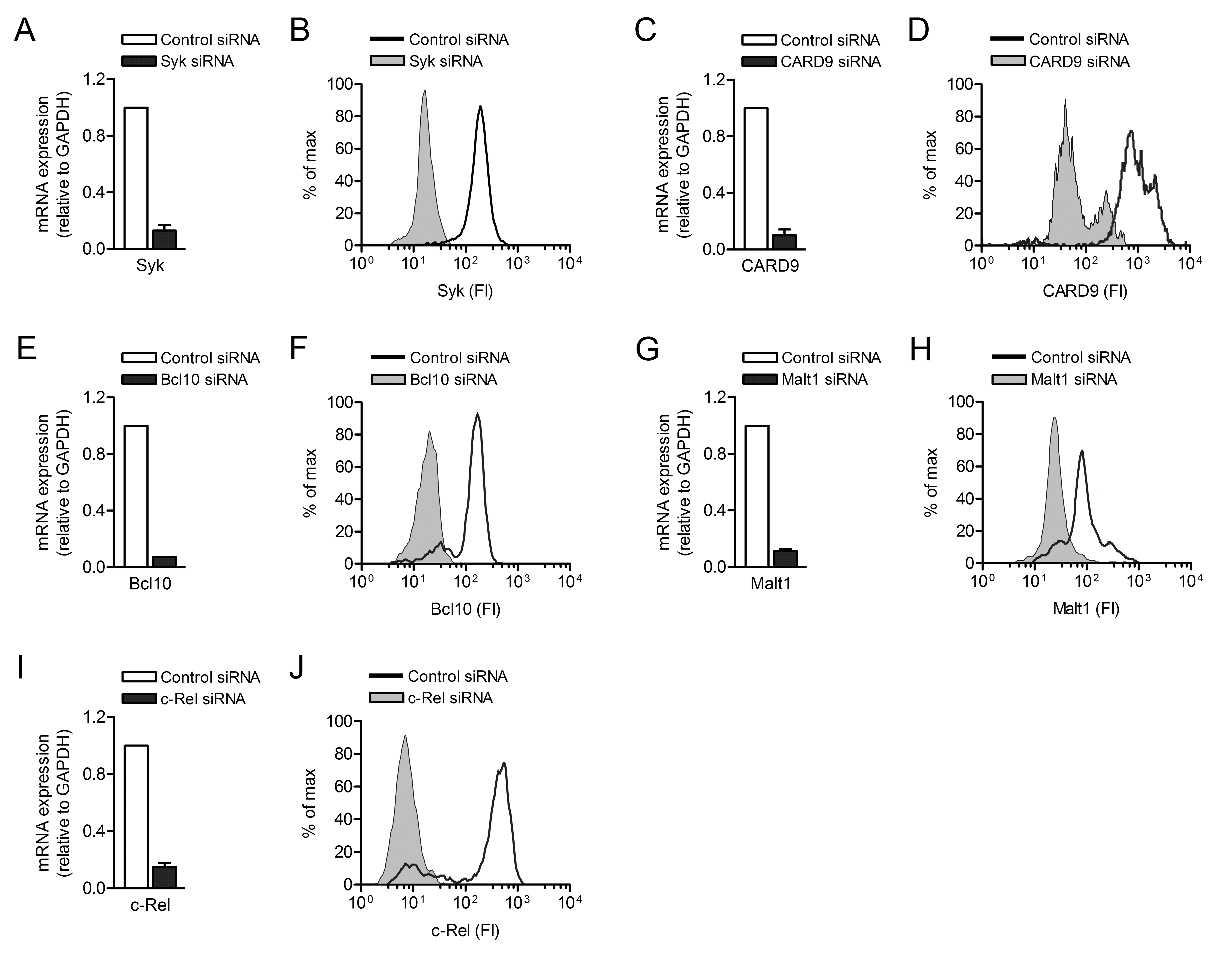

Supplement: Figure S1 — Silencing of Syk, CARD9, Bcl10, Malt1 and c-Rel in human primary DCs by RNA interference. Indicated proteins were silenced using specific SMARTpools, and non-targeting siRNA as a control. Silencing was confirmed by quantitative real-time PCR (A, C, E, G and I), or by staining and flow cytometry (B, D, F, H and J). In (A, C, E, G and I), expression is normalized to GAPDH and set at 1 in control siRNA-treated cells. Data are mean ± s.d. of at least four independent experiments (A, C, E, G and I) or are representative of at least two independent experiments (B, D, F, H and J). (2.24 MB TIF) [file ppat.1001259.s001.tif]

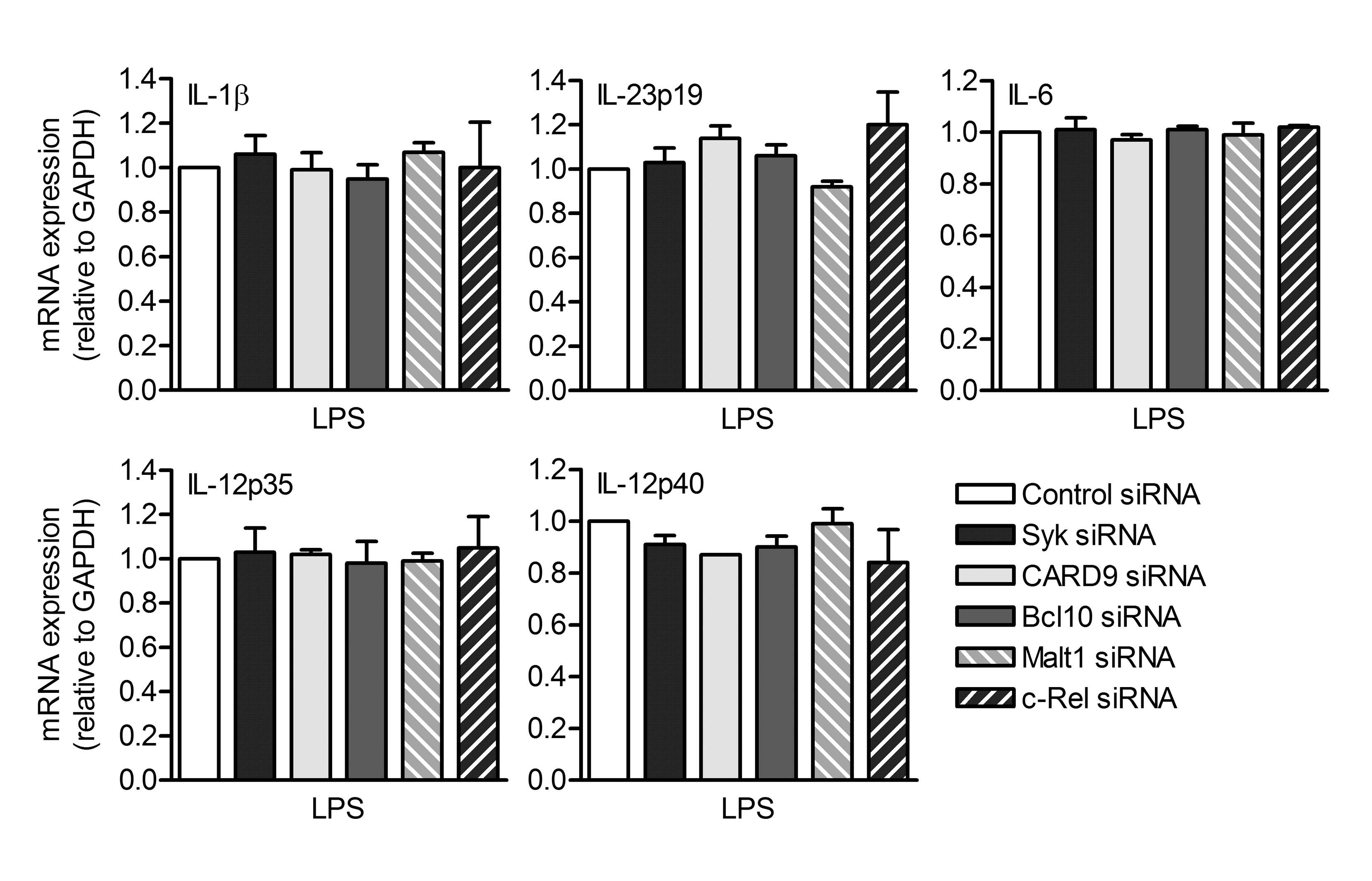

Supplement: Figure S2 — LPS signaling is not affected by Syk, CARD9, Bcl10, Malt1 and c-Rel silencing. Quantitative real-time PCR of indicated mRNAs in curdlan-stimulated DCs after Syk, CARD9, Bcl10, Malt1 and c-Rel silencing by RNA interference (siRNA). Expression is normalized to GAPDH and set at 1 in curdlan-stimulated cells. Data are mean ± s.d. of at least three independent experiments. (2.22 MB TIF) [file ppat.1001259.s002.tif]

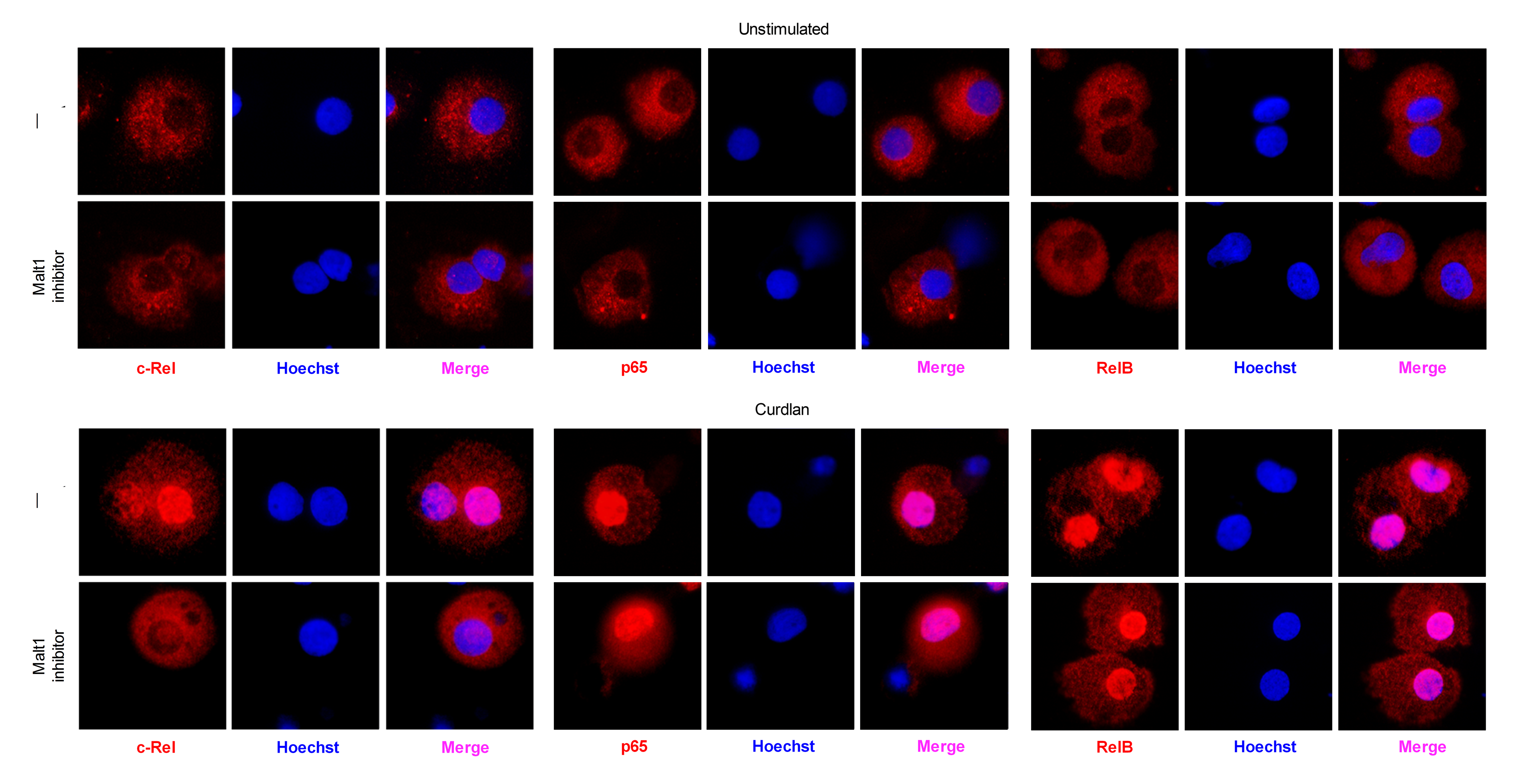

Supplement: Figure S3 — Malt1 paracaspase activity is required for c-Rel activation by dectin-1. Translocation of c-Rel, p65 or RelB (red) into the nucleus (Hoechst staining, blue; colocalization (Merge, pink)) in curdlan-stimulated DCs after Malt1 paracaspase inhibition by z-VRPR-FMK. Stainings are representative of two independent experiments. (7.54 MB TIF) [file ppat.1001259.s003.tif]

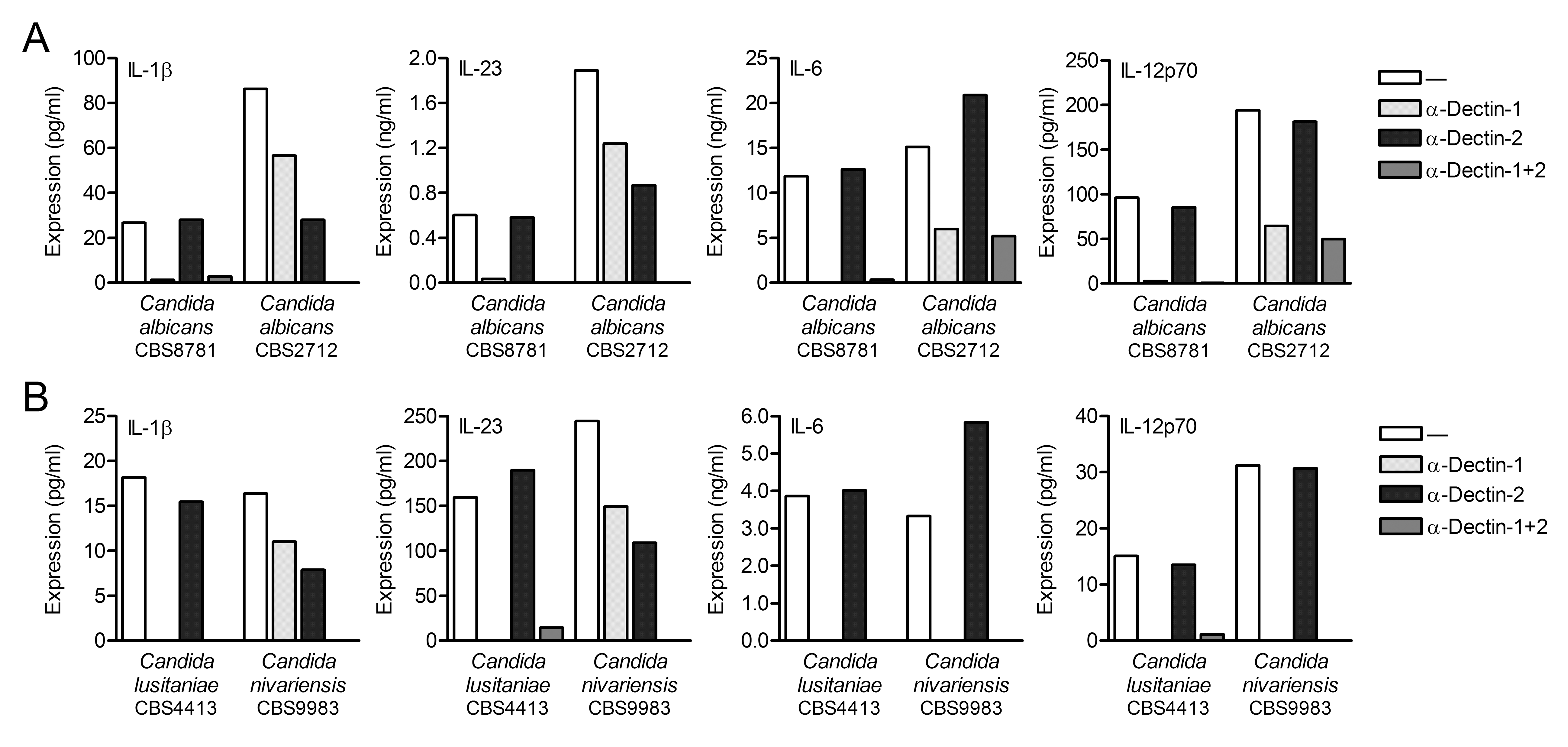

Supplement: Figure S4 — Dectin-1 and dectin-2 contribute to Candida spp.-induced cytokine expression. Cytokine production was determined by ELISA in supernatants of DCs stimulated with Candida albicans spp. (A), C. nivariensis or C. lusitaniae (B) in the absence or presence of blocking antibodies against dectin-1 and/or dectin-2. Data are representative of two independent experiments. (2.36 MB TIF) [file ppat.1001259.s004.tif]
