# Supplementary material for: Selective C-Rel Activation via Malt1 Controls Anti-Fungal TH-17 Immunity by Dectin-1 and Dectin-2
Source: PLoS Pathog. 2011 Jan 20;7(1):e1001259. doi: 10.1371/journal.ppat.1001259 (PMC3024268; doi:10.1371/journal.ppat.1001259)
Supplement: Table S1 — Expression primer sequences. (0.05 MB DOC) [file ppat.1001259.s005.doc]

Table S1

**Expression primer sequences**

| **Gene product** | **Forward primer** | **Reverse primer** |
| --- | --- | --- |
| IL-1β | TTTGAGTCTGCCCAGTTCCC | TCAGTTATATCCTGGCCGCC |
| IL-23p19 | GCTTGCAAAGGATCCACCA | TCCGATCCTAGCAGCTTCTCA |
| IL-6 | TGCAATAACCACCCCTGACC | TGCGCAGAATGAGATGAGTTG |
| IL-12p35 | CTCCAGAAGGCCAGACAAAC | AATGGTAAACAGGCCTCCACT |
| IL-12p40 | CCAGAGCAGTGAGGTCTTAGGC | TGTGAAGCAGCAGGAGCG |
| CARD9 | CATGTCGGACTACGAGAACGAT | CAGGTAAGGTGTGATGCGTGA |
| Bcl10 | ATGGAGCCACGAACAACCTCT | TCGTGCTGGATTCTCCTTCTG |
| Malt1 | GACCCATTCCATGGTGTTTACC | AATAAATGCATCTGGAGTCCGG |
| Syk | CCAGAGACAACAACGGCTCC | TGTCGATGCGATAGTGCAGC |
| c-Rel | CTGGCCAACATGGTGAAACC | CAGGCACGTACCACCATGC |
| GAPDH | CCATGTTCGTCATGGGTGTG | GGTGCTAAGCAGTTGGTGGTG |

**ChIP primer sequences**

| **Gene product** | **Forward primer** | **Reverse primer** |
| --- | --- | --- |
| IL-1β NF-κB | CTGTGTGTCTTCCACTTTGTCCC | TGCATTGTTTTCCTGACAATCG |
| IL-23p19 NF-κB  (site 1) | CATCCCAGGCCTCTAGCC | GGTTTGGTTCCCTCAGTTGTTC |
| IL-6 NF-κB | ACGACCTAAGCTGCACTTTTCC | GAGCCTCAGACATCTCCAGTCC |
| IL-12p35 NF-κB | GAGTACTCAGCCCGCCAGG | CCTCTTTGCAGGAGACGGC |
| IL-12p40 NF-κB | TCTTGAAATTCCCCCAGAAGG | GGACGGAGAGTCCAATGGC |
| GAPDH RNAPII | TACTAGCGGTTTTACGGGCG | TCGAACAGGAGGAGCAGAGAGCGA |
